# A Comparative Clinical Evaluation of Effectiveness of Platelet-Rich Plasma, Synthetic Allograft, and Bioresorbable Xenograft During Immediate Implant Placement

**DOI:** 10.7759/cureus.32121

**Published:** 2022-12-01

**Authors:** Nitin Tomar, Surya Dahiya, Pradeep Kumar Sharma, Ipe Sabu K, Anuj Singh Parihar, Ananyo Mandal, Amiya Ranjan Sahoo

**Affiliations:** 1 Department of Public Health Dentistry, People's Dental Academy, Bhopal, IND; 2 Department of Conservative Dentistry and Endodontics, Maharishi Markandeshwar College of Dental Sciences and Research (MMCDSR), Mullana, IND; 3 Department of Oral Surgery, Government Dental College, Dibrugarh, IND; 4 Department of Prosthodontics and Crown and Bridge, PSM College of Dental Science and Research, Thrissur, IND; 5 Department of Periodontics, People’s Dental Academy, Bhopal, IND; 6 Department of Conservative Dentistry and Endodontics, Kusum Devi Sunderlal Dugar Jain Dental College, Kolkata, IND; 7 Department of Oral and Maxillofacial Surgery, Awadh Dental College and Hospital, Jamshedpur, IND

**Keywords:** prp, clinical efficacy., bone formation, osseointegration, allograft, xenograft, implant, immediate

## Abstract

Background: The use of an appropriate graft material helps in an adequate amount of osseointegration. The objective of this study was to compare the efficacy of platelet-rich plasma (PRP), a synthetic allograft (PerioGlas), and Bio-Oss, a bioresorbable xenograft in immediate implant procedures.

Methods: In this randomized, prospective study, 90 patients were categorized into three groups with 30 samples in each as Group A: patients who received PRP with an immediate implant; Group B: immediate implants with synthetic allograft (PerioGlas); and Group C: patients with immediate implants placed using bioresorbable xenograft (Bio-Oss). Postoperative follow-up was done based on plaque and gingival index, measurement of probing depths, and resorption of bone. Obtained data were statistically analyzed using the "one-way analysis of variance (ANOVA)" test.

Results: Inter-group statistical comparisons between gingival and plaque indices at three, six, and 12 months of follow-up in the study groups demonstrated no statistical significance (p > 0.05). The mean probing depths and resorption of bone at three, six, and 12 months of follow-up were statistically nonsignificant (p > 0.05) on the inter-group comparison.

Conclusion: It could be concluded from the present study that there is no statistical superiority observed among PRP, PerioGlas, or Bio-Oss in terms of their usage as a graft material along with immediate implant placement procedure.

## Introduction

Platelet-rich plasma (PRP) is an autogenously generated platelet concentrate within a certain plasma volume. PRP is an abundantly rich source of autogenously found growth-based factors [1]. Traditionally allografts have been obtained from donors belonging to identical species. These are available as fresh or frozen, freeze-dried osseous, and de-mineralized freeze-dried osseous tissues [2]. These allograft materials act as osteo-conductive scaffolds with osteo-inductive properties due to proteins, for example, bone morphogenetic protein [3]. On the other hand, Xenografts are derived from different species [1].

PRP can be used either alone or as a combination with autograft as well as allograft materials for treating periodontal bony defects, preservation of extraction sockets, augmentation of the alveolar bony ridge, reconstruction of the mandible, elevation of the floor of the sinus, and repair of maxillary clefts [3]. Plachokova et al. (2008) have demonstrated higher volume as well as denser bones when compared with the use of autografts alone for regenerating bones [4]. It has been found that PRP is synthesized from the host and is rich in various growth factors that help in bone formation and the healing process.

Autologous concentrates of platelets such as PRP or platelet-rich fibrin are easily available, cost-effective, and contain high concentrations of a variety of growth factors that have a role in the healing as well as regeneration of tissues [5].

Various bioactive growth factors that get released on activation of platelets are platelet-derived growth factor, transforming growth factor, vascular endothelial growth factor, insulin-like growth factor along with epidermal growth factor. These growth proteins have histopromotive properties, which play a role in the healing of tissues, chemotaxis, cellular proliferation as well as tissue differentiation, removal of tissue-derived debris, angiogenesis as well as production of extracellular matrix [6-8].

Bio-Oss, a xenograft, has high porous structural composition, which acts by stimulating greater numbers of capillaries during the growth, proliferation, and migration of osteoblasts. On the other hand, PerioGlas (an allograft) acts by promoting osteo-conduction and its capability for binding to soft as well as osseous tissues. This property deals with wide popularity when compared with any other alloplastic graft materials [1-3].

At the time when dental implant placement is done within a fresh tooth extraction socket, there is a creation of some space between the periphery of an implant and the surrounding cortical bone. This gap may be created over any of the aspects of an immediate inserted implant, i.e., buccally, lingually, and/or proximally. The space created between the periphery of an implant and the surrounding bone is termed a "gap" or "jumping" distance. Bone filling within this jumping distance between the immediately placed implant as well as the peripherally present bone is crucial [9]. Specifically, the buccal side of an immediately placed implant has maximum importance as it comprises the esthetic zone, and also the buccal bone plate is thinner and its resorption may cause recession of soft tissue [9-11].

Hence, keeping in mind the creation of gap or jumping distance during the immediate placement of implants, the aim of this study was to compare the clinical efficacy of PRP, synthetic allograft, and bioresorbable xenograft materials following immediate implant placement.

## Materials and methods

Study design

This was a prospective, randomized controlled clinical trial for which a total of 90 patients were enrolled after obtaining written informed consent from the participants. The selected patients were grouped into three categories with 30 samples in each as Group A: patients who received PRP following immediate implant placement; Group B: immediate implants placed along with synthetic allograft; and Group C: placement of immediate implants done using bioresorbable xenograft. The study was conducted from February 2018 to March 2020.

Ethical approval

Ethical approval was obtained from the institutional ethics committee of People’s Dental Academy with IEC Ref No. PDA/Dean/2018/781-02.

Sample size calculation

The sample size for this study was calculated based on the following formula:

 n = Z^2^_a/2_ pq/d^2^

= 1.96 * 1.96 * 0.30 * 0.70/(0.10 * 0.10) = 80.67,

where p is the observed incidence of patients fulfilling the requirement for immediate implant placement, q = 1 - p, d is the margin of error, and Z^2^_a/2_ is the ordinate of standard normal distribution at α% level of significance. A sample size of 80.67 was obtained from the formula and it was increased to 90.

Subject inclusion criteria

a) Adequate oral hygiene, b) sufficient volume of bone for placing the implant (i.e., the thickness of bone labially measuring 2 mm or greater and minimum 5 mm apically present bone which was measured using cone beam computerized tomography (CBCT) and gap or jumping distance measuring less than 2 mm), and c) patients' age in the range of 30-55 years.

Exclusion criteria

a) Those who had any systemic disorder or disease condition that could cause impairment of bone healing, b) the presence of any infection and/or periodontal disease or bone dehiscence or an absence of bony cortical plate following extraction of a tooth, c) patients with any physical and/or mental impairment, d) patients who smoke heavily, and d) those participants incapable of following appointments for follow‑up.

Statistical analysis

Statistical tests were conducted using the statistical software Statistical Package for Social Science (SPSS) system version 21.0 (IBM Corp, Armonk, NY, USA). Continuous variables were observed as mean values for non-normally distributed data. The p-value was calculated using a one-way analysis of variance (ANOVA) test. Significance was set below 0.05.

Demographic data of each patient were recorded along with a detailed history. Clinical and radiographic findings were noted before the procedure. In all cases, implants were placed following standardized clinical and laboratory protocols.

Technique for PRP preparation

The two-milliliter volume of PRP gel was prepared on the same day of performing the surgical procedure. For this purpose, 10 mL of venous blood was drawn from a particular patient and was collected in a 10 mL sterile Vacutainer tube, which contained 0.5 mL of sodium citrate. These loaded Vacutainer tubes were centrifuged by the use of a centrifugation machine (REMI 2000) at 200 × g for 20 minutes. Separated plasma was then transferred in a 15 mL conical sterile polypropylene tube which was centrifuged at a speed of 400 × g for a total duration of 10 minutes. The top portion of the resultant sample consisted of "platelet-poor" plasma while the lower portion contained "platelet-rich" plasma. The lower portion was carefully separated and transferred to a sterile glass Dappen dish as a PRP for usage.

Commercially available PerioGlas, a synthetic allograft, and a bioresorbable xenograft material, Bio-Oss, were used as bone grafting materials in Groups B and C, respectively.

Surgical protocol

All surgical procedures were performed by a trained surgeon under adequate local anesthesia and aseptic conditions. Indicated teeth were luxated carefully and were then extracted using extraction forceps atraumatically. Any granulation tissue, if present, was then removed from the extraction sockets which were then prepared for placement of dental implants The sockets were then further evaluated on a CBCT scan to decide about the dimensions of the implant to be placed. Implants were placed after reflecting full-thickness mucoperiosteal flaps along with vertical releasing incisions and palatal flaps were reflected to expose the crest to provide visualization of the buccal and lingual bone plates. A surgical pouch was prepared on the buccal/labial side with the help of a periosteal elevator [12].

The axis of the implant was positioned parallel to the occlusal forces. To prevent excessive heat generation, the drill was used in a reduction gear handpiece with a physio-dispenser. Drilling was done in succession at 800-1000 rpm until the desired dimensions were reached. Implant platforms were placed at the level of the alveolar crestal bone followed by the cover screw placement. Placement of implants of varying sizes (i.e., length measured between 10 and 14 mm; diameters measured between 3.4 and 3.8 mm; Friadent, Dentsply) was done as per the standard procedure. In patients, with nil or loss of labial bony plate, crestal bone level was determined using a periodontal probe. A bone graft was placed in the created surgical pouch of sockets with either PRP, synthetic allograft material (PerioGlas), or xenograft (Bio‑Oss), respectively, in Groups A, B, and C. The membrane was placed over the graft, and suturing was done with 3‑0 vicryl sutures to close the surgical wound. Oral hygiene instructions were given to the patients and were followed up for three, six, and 12 months for clinical and CBCT evaluation.

Plaque and gingival index and probing depth assessment

The soft tissue was evaluated using a modified plaque index, gingival index [13], and probing depth at four sites (mesial, distal, buccal, and lingual) at three, six, and 12 months of follow-up. A calibrated probe was used to calculate the probing depth at the immediate implantation site and the full-mouth site.

CBCT imaging

CBCT evaluation was done for each patient at baseline, three months, six months, and 12 months of the follow-up period. The amount of bone resorption was measured from the crestal bone level to the implant crest module at the mesial and distal sites. Alterations in the width of the labial cortical plate were determined by measuring from the outermost labial implant surface or cortical bone till the outline of the implant fixture at four levels at the alveolar bony crest or the implant platform which were 2 mm as well as 6 mm below the alveolar bone crest and at the level of the apical portion of the placed implant.

All CBCT images were made by using an i-CAT-CBCT scanner (Imaging Sciences International Pvt Ltd.) at 120 kV, 37.07 mA, 0.2 Voxel size (mm^3^), 13x16 FOV (H × W) (cm), exposure time 26.9 seconds. 

All CBCT images were reconstructed using digital image software and language communication in a medicine language format. All reconstructed images were processed by using "HU iCAT Vision Q" software.

Assessment for implant success

The following criteria were used to determine the implant's success: first, the absence of any clinically or radiographically detectable signs of peri-implant inflammation or infection at the implant site; and second, the absence of clinical mobility.

## Results

Gingival and plaque index scores

Gingival indices that were measured after implant placement at a follow-up of three months demonstrated a score of 0.64 in Group A, 0.76 in Group B, and 0.67 in Group C, while at a six-month follow-up, the gingival index was observed as 0.59 in Group A, 0.69 in Group B, and 0.61 in Group C and subsequently at follow-up of one year, the gingival index was found to be 0.54 in Group A, 0.65 in Group B, and 0.59 in Group C. Inter-group comparison showed no significant statistical difference (p > 0.05) (Table 1).

**Table 1:** Comparison between gingival indices at different follow-up intervals in all three groups p < 0.05. SD, standard deviation.

Parameters	Duration	Group A		Group B		Group C		p-Value
		Mean	SD	Mean	SD	Mean	SD	
Gingival index (at the site of implant placement)	3rd month	0.64	0.13	0.76	0.12	0.67	0.12	0.07
	6th month	0.59	0.12	0.69	0.11	0.61	0.12	0.09
	12th month	0.54	0.14	0.65	0.13	0.59	0.11	0.08

On the other hand, inter-group comparison in plaque index score demonstrated no statistically significant difference (p > 0.05) (Table 2).

**Table 2:** Comparison between plaque index at subsequent follow-ups in all study groups p < 0.05. SD, standard deviation.

Parameters	Duration	Group A		Group B		Group C		p-Value
		Mean	SD	Mean	SD	Mean	SD	
Plaque index (at implant site)	3rd month	0.71	0.14	0.73	0.13	0.69	0.13	0.09
	6th month	0.69	0.15	0.71	0.12	0.70	0.11	0.08
	12th month	0.68	0.13	0.62	0.11	0.59	0.12	1.0

Probing depth assessment

No statistically significant differences were observed in any of the study groups on assessing the probing depths on designated follow-up intervals of three, six, and 12 months (p > 0.05) (Table 3). 

**Table 3:** Mean probing depths observed at three-, six-, and 12-month follow-up in the studied groups on buccal, lingual, mesial, and distal aspects p < 0.05. SD, standard deviation.

Follow-up	Site	Group A		Group B		Group C		p-Value
		Mean	SD	Mean	SD	Mean	SD	
3rd month	Mesial	2.41	0.24	2.36	0.25	2.34	0.26	0.52
	Distal	2.22	0.23	2.21	0.26	2.18	0.24	0.65
	Buccal	2.20	0.25	2.22	0.24	2.17	0.24	0.82
	Lingual	2.32	0.25	2.26	0.23	2.18	0.25	0.68
6th month	Mesial	2.39	0.23	2.34	0.24	2.32	0.24	0.54
	Distal	2.12	0.25	2.19	0.25	2.15	0.25	0.67
	Buccal	2.19	0.24	2.18	0.23	2.17	0.25	0.84
	Lingual	2.31	0.24	2.24	0.22	2.16	0.23	0.71
12th month	Mesial	2.22	0.25	2.11	0.23	2.09	0.23	0.76
	Distal	2.15	0.23	2.17	0.25	2.18	0.24	0.34
	Buccal	2.25	0.24	2.23	0.24	2.22	0.23	0.89
	Lingual	2.03	0.24	2.04	0.23	2.04	0.24	0.56

Assessment of bone resorption

No statistically significant differences (p > 0.05) were observed in observing mean levels of bone resorption in all aspects at follow-up intervals (Table 4).

**Table 4:** Mean bone resorption in Groups A, B, and C at 12-month follow-up p < 0.05. SD, standard deviation.

Follow-up	Bone resorption (in mm)							p-Value
	Aspect	Group A		Group B		Group C		
		Mean	SD	Mean	SD	Mean	SD	
3rd month	Mesial	5.01	0.32	4.87	0.35	4.89	0.34	0.18
	Distal	4.87	0.34	4.07	0.33	4.23	0.36	0.17
6th month	Mesial	3.45	0.41	3.39	0.34	3.37	0.40	0.29
	Distal	3.69	0.38	3.65	0.37	3.59	0.32	0.26
12th month	Mesial	3.09	0.32	3.12	0.38	3.15	0.34	0.74
	Distal	3.25	0.36	3.16	0.35	3.19	0.36	0.19

## Discussion

A dental implant serves as an excellent means of bone preservation and replacement of edentulous tooth or teeth. An immediate implant is highly popular nowadays and is extremely successful if used with appropriate grafting material. Fresh tooth extraction sockets are enriched with freshly exposed periodontal cells and tissues and can support quick bone growth, healing, and reduction in bone resorption.

A graft material is typically placed in the area between a dental implant and the wall of the socket for promoting the process of osseo-integration. An autogenous type of bone graft is considered to possess the best material properties related to osteo-inductiveness, osteo-conductiveness, and osteogenetic nature [11].

In the current study, none of the graft materials, i.e, PRP, PerioGlas (a synthetic allograft material), and Bio-Oss (a bioresorbable xenograft), were found to be statistically superior to another in terms of clinical indices observed, i.e., probing depth and mean bone resorption during the follow-up intervals. Our study findings are in contrast with the observations made by Daniel et al. who found that both PerioGlas, as well as Bio-Oss graft materials in the site of immediately placed implant, indicated excellent osseointegration surrounding a site of immediate placement of implant [12].

Similarly, Viswambaran et al. (2014) reported no implant failure at a three-month follow-up period in the sample of patients after placing implants with freeze-dried bone allograft as well as modified hydroxyapatite [14].

In accordance with our findings, Goutam et al. (2022) also observed no statistically significant difference between the Bio-Oss and PerioGlas graft materials during immediate implant placement [15].

Hence, on analyzing the primary outcome of this study, it was observed that none of the graft materials used, i.e., PRP, Bio-Oss, or PerioGlas, demonstrated superior quality in new bone formation or osseointegration properties over each other. Hence, the secondary outcome of this study can be the cost-effectiveness involved during the placement of immediate implants with equal clinical and biological efficacy. Single-step implant placement procedure along with adjunct use of PRP increases the ability of healing of peri-implant tissue. This helps in creating favorable quality of soft as well as hard tissue inter-relationships [16,17]. Additionally, there is an additional advantage accompanied by psychological boosting of a patient by providing a fixed tooth replacement within a very less period of time [18-20].

There are reported studies that compared only PerioGlas (synthetic allograft) and Bio-Oss (bioresorbable xenograft material). Daniel et al. [12] from their study found that both synthetic allograft and bioresorbable xenograft are promising and equally potential in bone formation around the immediate implant site. The results are similar to our findings. But there are no reported studies along with PRP. Hence, the present study is unique, which compared PerioGlas, Bio-Oss, and PRP with CBCT evaluation.

In the current research, since all three materials showed the same results, the material whichever is readily available and economical can be used. PRP is freshly prepared from the patients; hence, it is less expensive and can be easily prepared while PerioGlas (synthetic allograft) (Group B) 2 x 0.5 cc is available at around 30 USD and Bio-Oss (bioresorbable xenograft material) (Group C) is available at around 97 USD for 0.5 g (1.5 cc).

Limitations

However, the results of the study can be better reflected if a larger sample size is used, which was a drawback of this study. Hence, further research in implant science can be advocated if the sample size is increased. 

## Conclusions

Dental implants are becoming increasingly popular nowadays due to quicker replacement of edentulous areas though this procedure works best if an appropriate graft material is used along with the implant to fill in the gap or jumping distance that exists between an implant surface and wall of the tooth extraction socket. In the present study, the investigators found no difference between PRP, Bio-Oss (a synthetic allograft material), and PerioGlas (a bioresorbable xenograft material). 

## References

[1] Sheikh Z, Hamdan N, Ikeda Y, Grynpas M, Ganss B, Glogauer M (2017). Natural graft tissues and synthetic biomaterials for periodontal and alveolar bone reconstructive applications: a review. Biomater Res.

[2] Lee MJ, Kim BO, Yu SJ (2012). Clinical evaluation of a biphasic calcium phosphate grafting material in the treatment of human periodontal intrabony defects. J Periodontal Implant Sci.

[3] Eppley BL, Pietrzak WS, Blanton MW (2005). Allograft and alloplastic bone substitutes: a review of science and technology for the craniomaxillofacial surgeon. J Craniofac Surg.

[4] Plachokova AS, Nikolidakis D, Mulder J, Jansen JA, Creugers NH (2008). Effect of platelet‐rich plasma on bone regeneration in dentistry: a systematic review. Clin Oral Implants Res.

[5] Marx RE, Carlson ER, Eichstaedt RM, Schimmele SR, Strauss JE, Georgeff KR (1998). Platelet-rich plasma: growth factor enhancement for bone grafts. Oral Surg Oral Med Oral Pathol Oral Radiol Endod.

[6] Einhorn TA (2005). The science of fracture healing. J Orthop Trauma.

[7] Alsousou J, Thompson M, Hulley P, Noble A, Willett K (2009). The biology of platelet-rich plasma and its application in trauma and orthopaedic surgery: a review of the literature. J Bone Joint Surg Br.

[8] Botticelli D, Berglundh T, Buser D, Lindhe J (2003). The jumping distance revisited: an experimental study in the dog. Clin Oral Implants Res.

[9] Cutrim ES, Peruzzo DC, Benatti B (2012). Evaluation of soft tissues around single tooth implants in the anterior maxilla restored with cemented and screw-retained crowns. J Oral Implantol.

[10] Bhowal K, Ghosh S (2020). Guided bone regeneration in immediate dental implants - review. Int J Sci Res.

[11] ArRejaie A, Al-Harbi F, Alagl AS, Hassan KS (2016). Platelet-rich plasma gel combined with bovine-derived xenograft for the treatment of dehiscence around immediately placed conventionally loaded dental implants in humans: cone beam computed tomography and three-dimensional image evaluation. Int J Oral Maxillofac Implants.

[12] Daniel D, Shetty V, Jose J, Kumar AH, Santosh BS, Saikrrupa SP (2020). The efficacy of synthetic allograft and bioresorbable xenograft in immediate implant procedures: a comparative clinical study. J Dent Implant.

[13] Löe H (1967). The gingival index, the plaque index and the retention index systems. J Periodontol.

[14] Viswambaran M, Arora V, Tripathi RC, Dhiman RK (2014). Clinical evaluation of immediate implants using different types of bone augmentation materials. Med J Armed Forces India.

[15] Goutam M, Batra N, Jyothirmayee K, Bagrecha N, Deshmukh P, Malik S (2022). A comparison of xenograft graft material and synthetic bioactive glass allograft in immediate dental implant patients. J Pharm Bioallied Sci.

[16] Anand U, Mehta DS (2012). Evaluation of immediately loaded dental implants bioactivated with platelet-rich plasma placed in the mandibular posterior region: a clinico-radiographic study. J Indian Soc Periodontol.

[17] Glauser R, Rée A, Lundgren A, Gottlow J, Hämmerle CH, Schärer P (2001). Immediate occlusal loading of Brånemark implants applied in various jawbone regions: a prospective, 1-year clinical study. Clin Implant Dent Relat Res.

[18] Sommer M, Zimmermann J, Grize L, Stübinger S (2020). Marginal bone loss one year after implantation: a systematic review of different loading protocols. Int J Oral Maxillofac Surg.

[19] Arghami A, Simmons D, St Germain J, Maney P (2021). Immediate and early loading of hydrothermally treated, hydroxyapatite-coated dental implants: a 7-year prospective randomized clinical study. Int J Implant Dent.

[20] Kim WH, Lee JC, Lim D, Heo YK, Song ES, Lim YJ, Kim B (2019). Optimized dental implant fixture design for the desirable stress distribution in the surrounding bone region: a biomechanical analysis. Materials (Basel).

